# Effects of xylooligosaccharide supplementation on performance, intestinal morphology, antioxidant capacity and cecal microflora in weaning pigs

**DOI:** 10.3389/fvets.2025.1655379

**Published:** 2025-09-17

**Authors:** Wenfeng Ma, Mengmeng Gao, Xiaoli Zhang, Xiaohong Wu, Pei Mao, Wenna Fan, Lingping Zhao, Shitu Tan

**Affiliations:** College of Animal Science and Technology, Henan University of Science and Technology, Luoyang, Henan, China

**Keywords:** xylooligosaccharide, weaned pigs, growth performance, antioxidant, cecal microbe

## Abstract

Study aimed to investigate the effects of xylooligosaccharide (XOS) supplementation on growth performance, antioxidant capacity, and cecal microorganisms in weaned pigs. Forty-eight 28-day-old hybrid weaned pigs Duroc × (Landrace × Yorkshire) of similar body weight (7.62 ± 0.41 kg) and genetic background were randomly divided into control group (CON) and experimental group (XOS). The CON group was fed a basal diet, and the XOS group was fed a diet supplemented with 500 mg/kg XOS. The pretest period lasted for 7 days, and the test period lasted for 28 days. Compared with the CON group, the XOS group significantly reduced the feed-to-gain ratio by 5.14%, increased alkaline phosphatase activity in serum, and improved the apparent digestibility of dietary ether extract (EE) and calcium (Ca) in weaned pigs (*p* < 0.05). In addition, XOS supplementation increased the villus height of the duodenum by 9.69% and that of the ileum by 11.64%, and decreased the crypt depth of the jejunum by 21.37% (*p* < 0.05). XOS supplementation increased superoxide dismutase (SOD) activity in serum and ileum by 10.48 and 48.43%, respectively; increased catalase (CAT) activity in serum, liver, and spleen by 34.96, 29.55 and 90.36%, respectively; and decreased the malonaldehyde (MDA) content in liver and spleen of weaned pigs by 35.66 and 33.85%, respectively (*p* < 0.05). Compared with the CON group, the cecal microbial community diversity of weaned pigs with XOS supplementation significantly differed (*p* < 0.05). XOS supplementation reduced the relative abundance of *Proteobacteria* and *Spirochaetes* in the cecum of weaned pigs, and improved the relative abundance of *Lactobacillus* and *Ruminococcus* in the cecum of weaned pigs, but decreased the relative abundance of *Spirillaceae NK4A136*, *Prevoid group*, and *AC2044 group of Spirillaceae* (*p* < 0.05). In conclusion, the dietary inclusion of 500 mg/kg of XOS increased the growth performance, apparent digestibility, and intestinal morphology of weaned piglets, stimulated the proliferation of probiotic microorganisms, and improved the antioxidant capacity of pigs. The results of this study can provide a certain theoretical basis for the application of XOS in weaned piglets.

## 1 Introduction

Xylooligosaccharide (XOS) belongs to a kind of oligosaccharides (1). Due to the lack activity of xylanase in the intestine of animals, the XOS cannot be hydrolyzed by the digestive enzymes of the small intestine, but can be utilized as a carbon source by beneficial intestinal bacteria in hindgut (2). Studies have found that XOS can regulate the composition of intestinal microorganisms, and play an important role in improving the host intestinal barrier function and maintaining the homeostasis of the intestinal environment (2, 3). XOS is produced primarily from lignin-rich fiber feedstocks such as corn cobs, peach palm waste, cauliflower stems, wheat bran, and sugarcane bagasse by enzymatic and thermal lysis (4–8). Studies have shown that dietary XOS can promote nutrient absorption and utilization, prevent small intestinal villus atrophy, enhance immune and intestinal barrier function, and improve the growth performance of animals such as weaned pigs and broilers (9–11).

In recent years, some experiments have also found that the increase of intestinal probiotics can improve the antioxidant capacity of animals, but the specific mechanism is not clear (12). The dietary XOS supplementation has been demonstrated to contribute to the optimization of intestinal flora, so it can be used as one of the potential ways to improve the antioxidant capacity of animals (13). The XOS has remarkable stability and heat resistance under both acidic and alkaline conditions, so it can pass through the stomach and small intestine until the posterior intestinal stage, and can play a regulatory role in animals at low-dose supplementation (14).

At present, the majority of studies have primarily focused on the interaction between XOS and the intestinal microbiota, and limited research has been conducted on the effects of XOS on the antioxidant capacity, apparent nutrient digestibility and intestinal development in weaned piglets. Therefore, this study aimed to evaluate the effect for 500 mg/kg XOS supplementation on growth performance, serum biochemical parameters, nutrient digestibility, intestinal morphology, antioxidant capacity, and cecal microbiota in weaned piglets, so as to comprehensively evaluate the application effect of XOS in weaned piglets.

## 2 Materials and methods

### 2.1 Animals and experimental design

The experimental animals were selected from Henan Xinda Animal Husbandry Co., LTD. Forty-eight Duroc × (Landrace × Yorkshire) weaned pigs of 28 days of age with an initial body weight of 7.62 ± 0.41 kg were randomly divided into two groups with six replicates per group and four pigs per replicate (half male and half female). The control group (CON) was fed a basal diet (as shown in Table 1), and the experimental group (XOS) was fed 500 mg/kg XOS on the basis of the control group. The pretest period lasted for 7 days, and the formal test period lasted for 28 days. The temperature (26–28°C) and humidity (60–70%) of the pig house were strictly controlled during the feeding phase. The remaining feed in the trough was cleared and weighed at regular intervals every morning, and the trough was supplemented with feed at 07:00, 12:00, and 18:00 every day. The piglets were given a feeding strategy of ad libitum feeding and drinking. The pig house was cleaned regularly to ensure dry hygiene.

**Table 1:** Composition and nutrient levels of the basal diet (air-dry basis, g/kg).

Ingredients	Content	Nutrient levels^b^	Content
Corn	376.3	Digestible energy (MJ/kg)	14.84
Extruded corn	156.2	Crude protein (%)	18.02
Soybean	32.5	Calcium (%)	0.80
Extruded soya	118.2	Ether extract (%)	5.25
Fish meal	34.3	Lys (%)	1.21
Wheat flour	68.7	Met (%)	0.38
Dried whey	69.0	Thr (%)	0.93
Fermented soybean	50.4	Trp (%)	0.25
Choline chloride (50%)	40.0	Met+Cys (%)	0.68
Glucose	49.4		
premix ^a^	5.0		
XOS ^c^	0/0.5		

^a^Provided per kilogram of diet: VA 2700 IU, VD3 300 IU, VE 25 IU, VK3 0.5 mg, VB1 1.50 mg, VB2 5.25 mg, VB12 0.026 mg, folic acid 0.3 mg, nicotinic 22 mg, pantothenate 14 mg, biotin 0.05 mg, Cu 5.6 mg, Fe 90 mg, Mn 5.2 mg, Zn 106 mg, Se 0.3 mg, I 0.2 mg.

^b^Digestible energy was the calculated value, and the other nutrient levels were analyzed value.

^c^Content of XOS: XOS (polymerized from 2 to 9 xylose molecules, 23%), cellulose (51%), hemicellulose (26%). XOS was supplemented at 0 (CON group), and 500 mg/kg by the replacement of the same amount of corn.

### 2.2 Growth performance

Feed consumption was recorded daily, and initial weight of each piglet was recorded at the beginning of the experiment. The final weight of each piglet was recorded at the end of the experiment. Average daily gain (ADG), average daily feed intake (ADFI), and feed to gain ratio (F/G) of piglets were calculated using the following formula. Piglets were weighed and fasted 12 h before slaughter and ensure normal water intake.


ADG=(final body weight—initial body weight)/days of feeding;

ADFI=total feed intake/(days of feeding×number of pigs).

### 2.3 Serum biochemical indicators

On the 28th day, a piglet randomly selected from each repeat for vena cava blood collected. After 10 min of standing, the serum was separated by using a table low-speed centrifuge (L500, Cence, Hunan, China) at 3,000 r/min for 10 min. The activities of serum alkaline phosphatase (ALP), aspartate aminotransferase (ALT) and alanine aminotransferase (AST) were measured by kits from Nanjing Jiancheng Bioengineering Institute (A059-2, C010-1, C009-2, respectively). Serum glucose (GLU), total protein (TP), total cholesterol (TC), triglyceride (TG), albumin (ALB), and globulin (GLB) were measured by automatic biochemical analyzer (BS-420, Mindray, Shenzhen, China).

### 2.4 Nutrient apparent digestibility

Fresh feces of piglets were collected by artificially stimulating their anal defecation for four consecutive days (days 24–27), with approximately 500 g of feces collected per pen per day. Sulfuric acid was added to fresh fecal samples to prevent nitrogen loss. The samples were treated under 65°C oven (LC-101-1, Lichen, Shanghai, China) 48 h, then return at room temperature for 24 h in the dryer. The dried samples were crushed and filtered through a 40-mesh sieve. The nutrient indicators of the samples were determined using the acid-insoluble ash method. Dry matter (DM), crude protein (CP), ether extract (EE), acid detergent fiber (ADF), neutral detergent fiber (NDF), calcium (Ca) phosphorus (P) and acid insoluble ash (AIA) was measured according to the methods described in Hu et al. (15). The apparent digestibility of nutrients was evaluated using the AIA method.


Nutrient apparent digestibility(%)=1−(A×D)/(B×C)×100.

A, content of the AIA in feed; B, content of the nutrient in fecal; C, content of the AIA in fecal; D, content of the nutrient in fecal.

### 2.5 Intestinal morphology

At the end of the experiment, one piglet was randomly selected from each replicate killed by a captive bolt and exsanguinated. The abdomen was opened to separate the intestine. The intestinal tissue specimens were fixed in 4% paraformaldehyde for 24 h and then stained. The hematoxylin–eosin (HE) staining kit (D006-1-4, Jiancheng Bioengineering Institute, Nanjing, China) was purchased from Nanjing Jian-Cheng Bioengineering Institute. The intestinal sections were scanned with the slice scanner (Pannoramic MIDI, 3dhistech, Budapest, Hungary), and the morphology of generated sections were measured using CaseViewer (Version 2.4, 3dhistech, Budapest, Hungary). More than 10 complete villi were selected from each section to measure the villus height (VH) and crypt depth (CD).

### 2.6 Antioxidant ability

The liver and spleen samples were collected at the central position of the tissues. The chyme of duodenum, jejunum and ileum was longitudinally rinsed with sterile saline, and the intestinal mucosal samples were gently scraped with a sterile dial. Superoxide dismutase (SOD) activity, hydrogen peroxide (CAT) activity and malondialdehyde (MDA) activity of these samples were then measured. The kits were purchased from Nanjing Jiancheng Bioengineering Institute (A001-3, A007-1 and A003-1).

### 2.7 Microbial diversity

The cecum was ligated at the location of anterior and posterior aspects, and then was cut open with a sterile scalpel. The cecum chymme was transferred to sterile cryotubes in a sterile environment, rapidly frozen in liquid nitrogen, and transferred to −80°C for 16srRNA sequencing of cecal microbiota. The total genomic DNA of the microbial community was extracted according to the instructions of the DNA extraction kit (T07-096, Majorbio, Shanghai, China), and the DNA purity and concentration were detected by agarose gel electrophoresis and ultramicro spectrophotometer (NanoDrop2000, Thermo, United States). The extracted target DNA was sequenced on the Illumina MiSep sequencing platform, and alpha diversity analysis, Veen plot analysis, beta diversity analysis, and species difference analysis were performed on the cloud platform (Majorbio, Shanghai, China).

### 2.8 Statistical analysis

The trial data were initially processed using Excel 2019 and then analyzed by independent sample t-test using SPSS 20.0 software. Standard error of mean (SEM) and mean were used to express the results, with *p* < 0.05 being a significant difference.

## 3 Results

### 3.1 Growth performance

Table 2 showed the effect of dietary supplementation of 500 mg/kg XOS on the growth performance of piglets from 0 to 28 d after weaning. Compared with the CON group, dietary supplemented with 500 mg/kg XOS significantly decreased the ADFI: ADG by 5.14% (*p* < 0.05).

**Table 2:** Effects of XOS on the growth performance of weaned pigs.

Items^1^	CON	XOS	SEM	*p*-value
Initial BW, kg^2^	7.67	7.57	0.29	0.82
Final BW, kg	20.58	21.04	0.78	0.69
ADG, g	464.00	482.00	10.41	0.27
ADFI, g	813.00	803.00	22.89	0.77
ADFI: ADG	1.75^a^	1.66^b^	0.02	0.04

^1^CON: basal diet, XOS = 500 mg/kg XOS + basal diet. Mean values within a same row between a and b were significantly different when *P* < 0.05, *n* = 6. ^2^BW, Body weight; ADG, Average daily gain; ADFI, Average daily feed intake.

### 3.2 Serum biochemical indicators

Table 3 showed the serum biochemical indicators of weaned pigs supplemented with XOS. Compared with CON group, the activity of serum ALP was significantly increased in weaned pigs with the addition of 500 mg/kg of XOS (*p* < 0.05).

**Table 3:** Effects of XOS on the serum biochemical indexes of weaned pigs.

Items	CON	XOS	SEM	*p*-value
TP, g/L^1^	48.57	51.13	2.42	0.46
ALB, g/L	26.51	28.73	1.71	0.62
GLB, g/L	22.07	22.43	1.19	0.66
ALT, U/L	87.01	94.12	4.12	0.25
AST, U/L	66.67	68.52	6.91	0.85
ALP, U/L	383.65^b^	459.25^a^	18.97	0.04
TC, mmol/L	2.18	2.11	0.27	0.84
TGs, mmol/L	0.47	0.43	0.05	0.54
GLU, mmol/L	9.57	8.68	0.69	0.33

^1^TP, Total proteins; ALB, Albumin; GLB, Globulin; ALT, Alanine transaminase; AST, Aspartate aminotransferase; ALP, Alkaline phosphatase; TC, Total cholesterol; TGs, Triglycerides; GLU, Glucose. Mean values within a same row between a and b were significantly different when *P* < 0.05, *n* = 6.

### 3.3 Nutrient apparent digestibility

As shown in Table 4, nutrient apparent digestibility of EE and Ca was significantly higher after the XOS supplementation to the diet compared to the CON group (*p* < 0.05).

**Table 4:** Effect of XOS on the apparent nutrient digestibility in weaned pigs.

Items	CON	XOS	SEM	*p-*value
DM, %^1^	89.55	90.18	0.27	0.45
CP, %	72.22	73.16	0.42	0.70
EE, %	73.87^b^	75.33^a^	0.13	0.03
NDF, %	50.87	51.63	0.72	0.70
ADF, %	35.15	35.58	0.17	0.82
Ca, %	59.72^b^	64.12^a^	0.44	<0.01
P, %	49.73	50.72	0.45	0.09

^1^DM, Dry matter; CP, Crude protein; EE, Ether extract; NDF, Neutral detergent fiber; ADF, Acid detergent fiber; Ca, Calcium; P, Phosphorus. Mean values within a same row between a and b were significantly different when *P* < 0.05, *n* = 6.

### 3.4 Intestinal morphology

As shown in Table 5, compared to the CON group, the addition of 500 mg/kg XOS to the diet significantly reduced the jejunal CD by 21.37% and increased the duodenal VH by 9.69% and ileal VH by 11.64% (*p* < 0.05).

**Table 5:** Effect of XOS on the intestinal morphology in weaned pigs.

Items	CON	XOS	SEM	*p-*value
Duodenum				
VH, μm^1^	513.11^b^	562.83^a^	12.35	0.04
CD, μm	263.67	210.11	22.07	0.16
VH/CD	2.01	2.69	0.22	0.09
Jejunum				
VH, μm	521.97	543.13	16.33	0.42
CD, μm	277.43^b^	218.14^a^	14.69	0.04
VH/CD	1.98	2.50	0.19	0.14
Ileum				
VH, μm CD, μm	495.63^b^	553.32^a^	12.86	0.03
	215.24	206.83	18.05	0.76
VH/CD	2.22	2.85	0.33	0.25

^1^VH, Villus height; CD, Crypt depth. Mean values within a same row between a and b were significantly different when *P* < 0.05, *n* = 6.

### 3.5 Antioxidant index

As shown in Table 6, the dietary addition of 500 mg/kg XOS increased the SOD activity in serum and jejunum of weaned piglets by 10.48 and 48.43%, respectively (*p* < 0.05). Meanwhile, the MDA content in the liver and spleen decreased by 35.66 and 33.85%, respectively (*p* < 0.05). And the CAT activity in the serum, spleen, and liver increased by 34.96, 29.55 and 90.36%, respectively (*p* < 0.05).

**Table 6:** Effect of XOS on antioxidant indexes in weaned pigs.

Items	CON	XOS	SEM	*p-*value
Serum				
SOD, U/mL^1^	337.31^b^	372.67^a^	5.82	0.02
MDA, nmol/mL	2.98	2.44	0.28	0.26
CAT, U/mL	3.89^b^	5.25^a^	0.33	0.04
Liver				
SOD (U/mg prot)	396.43	428.87	12.71	0.26
MDA (nmol/mg prot)	1.29^a^	0.83^b^	0.18	0.04
CAT (U/mg prot)	81.93^b^	106.14^a^	5.06	0.03
Spleen				
SOD (U/mg prot)	8.77	10.76	1.67	0.47
MDA (nmol/mg prot)	2.60^a^	1.72^b^	0.21	0.04
CAT (U/mg prot)	4.77^b^	9.08^a^	0.36	0.01
Duodenum				
SOD (U/mg prot)	75.31	84.46	5.15	0.24
MDA (nmol/mg prot)	0.46	0.22	0.14	0.34
CAT (U/mg prot)	1.70	2.29	0.19	0.11
Jejunum				
SOD (U/mg prot)	59.24^b^	87.93^a^	4.72	0.01
MDA (nmol/mg prot)	0.47	0.17	0.13	0.19
CAT (U/mg prot)	1.88	2.45	0.29	0.26
Ileum				
SOD (U/mg prot)	63.56	72.06	6.87	0.45
MDA (nmol/mg prot)	0.48	0.20	0.13	0.22
CAT (U/mg prot)	1.41	2.09	0.33	0.23

^1^SOD, Superoxide dismutase; MDA, Malondialdehyde; CAT, Catalase. Mean values within a same row between a and b were significantly different when *P* < 0.05, *n* = 6.

### 3.6 Microbial diversity in the cecum

#### 3.6.1 OTU horizontal Veen diagram analysis

**Figure 1:**
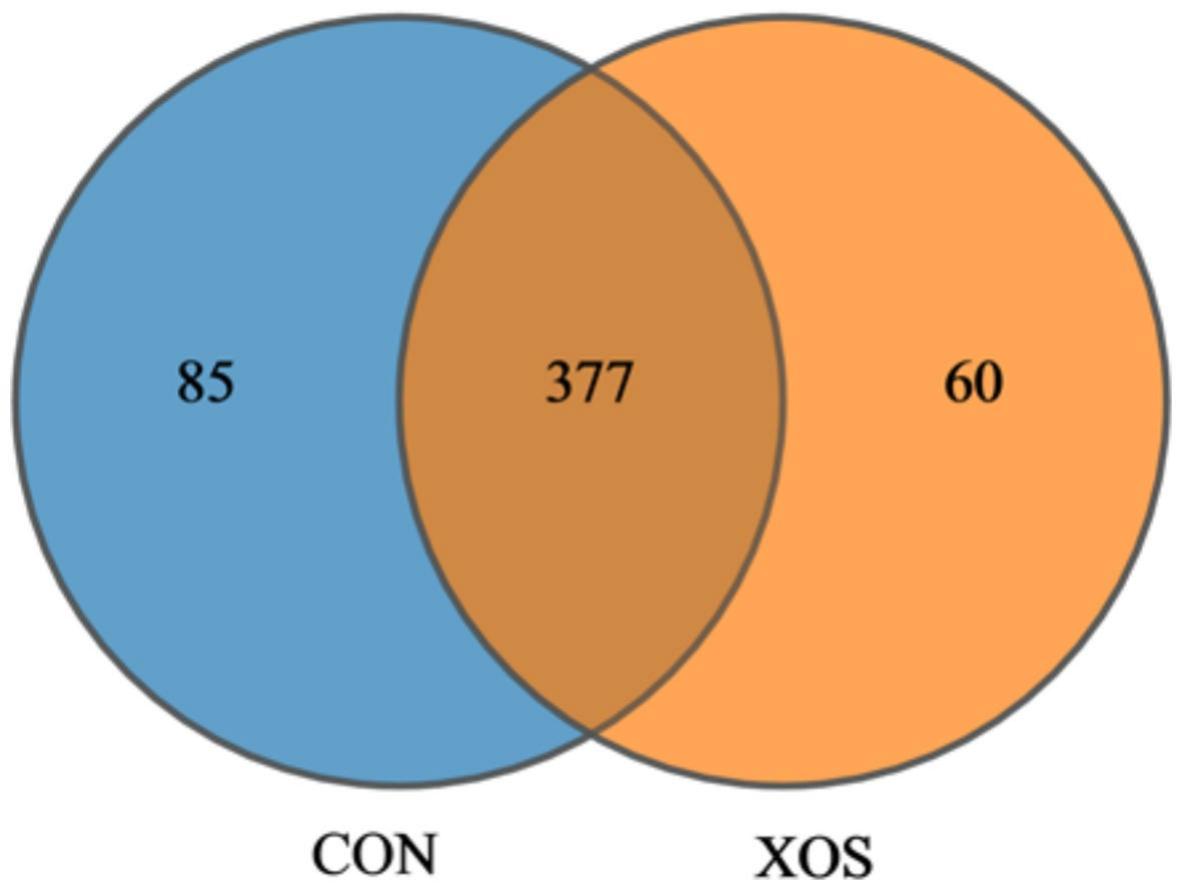
Venn diagram. CON, control treatment; XOS, 500 mg/kg XOS added to the diet on the basis of control treatment.

As shown in Figure 1, a total of 522 OTUs were detected in the cecum microbial of weaned pigs, with a total of 377 OTUs in the control and experimental groups, 85 OTUs specific to the control group and 60 OTUs specific to the experimental group.

#### 3.6.2 Beta diversity analysis

**Figure 2:**
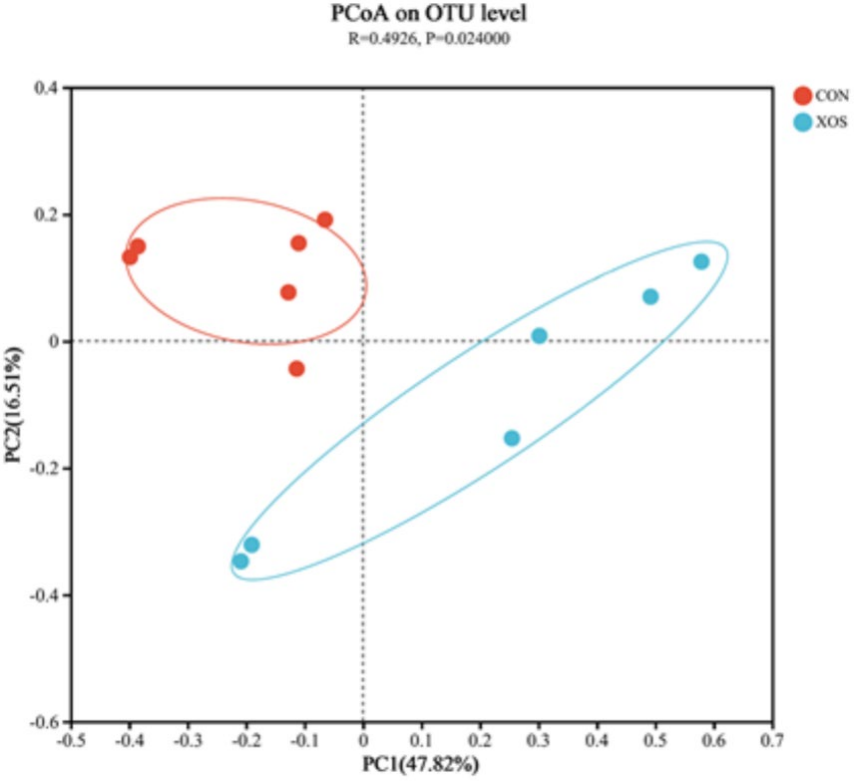
PCoA diagram (OTU level).

As shown in Figure 2, the caecal microbial community composition existed significant differences between the CON and XOS group by using the analysis of the PCoA (*p* < 0.05).

#### 3.6.3 Microbiome level composition of the cecum

**Figure 3:**
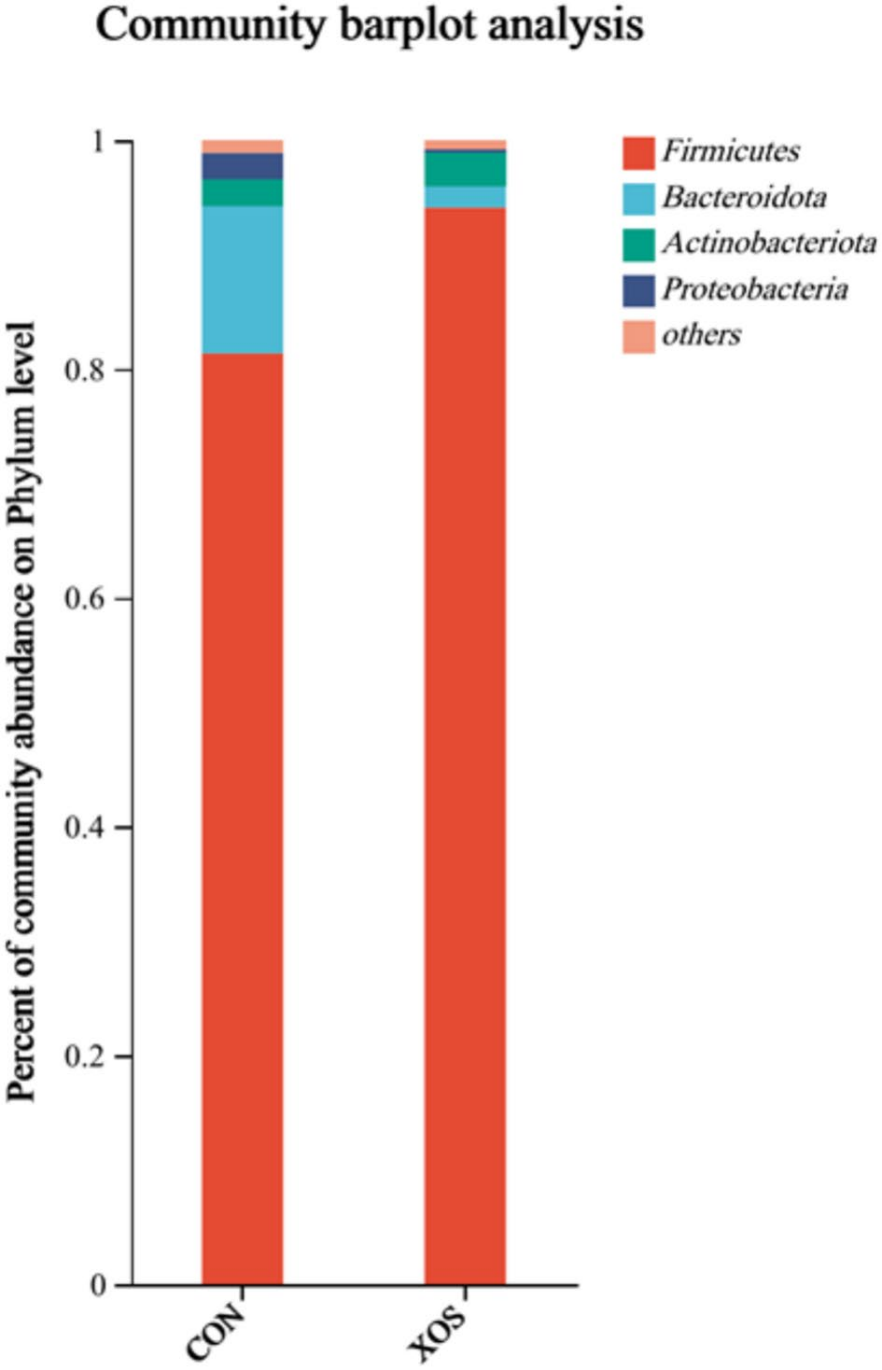
Effects of XOS on the composition of cecal microbial flora at phylum level in weaned pigs.

From Figure 3 and Table 7, a total of 12 phyla were identified, mainly consisting of four phyla, *Firmicutes*, *Bacteroides*, *Actinobacteria*, and *Proteobacteria*. The relative abundance of *Bacteroidetes* decreased significantly by 87.38% and that of *Proteobacteria* decreased significantly by 90.20% (*p* < 0.05). In addition, the relative abundance of *Spirochaetes* in non-major phyla was significantly reduced by 84.44% (*p* < 0.05).

**Table 7:** Effects of XOS on the composition of cecal microbial flora at phylum level in weaned pigs.

Items	CON	XOS	SEM	*p-*value
*Firmicutes*	81.36	91.89	8.21	0.17
*Bacteroidetes*	12.92^a^	1.63^b^	2.79	0.04
*Proteobacteria*	2.96^a^	0.29^b^	1.32	0.03
*Actinobacteriota*	2.34	2.82	0.22	0.45
*Patescibacteria*	0.59	0.29	0.24	0.24
*Spirochaetes*	0.45^a^	0.07^b^	0.19	0.04
*Verrucomicrobiota*	0.02	0.21	0.11	0.07
*Cyanobacteria*	0.02	0.10	0.01	0.07
*Unclassified*	0.01	0.01	0.01	0.59
*Desulfobacterota*	0.00	0.01	0.00	0.43
*Campilobacterota*	0.00	0.00	0.00	0.07
*Elusimicrobiota*	0.00	0.00	0.00	0.36

Mean values within a same row between a and b were significantly different when *P* < 0.05, *n* = 6.

#### 3.6.4 Microbial genus level composition of the cecum

**Figure 4:**
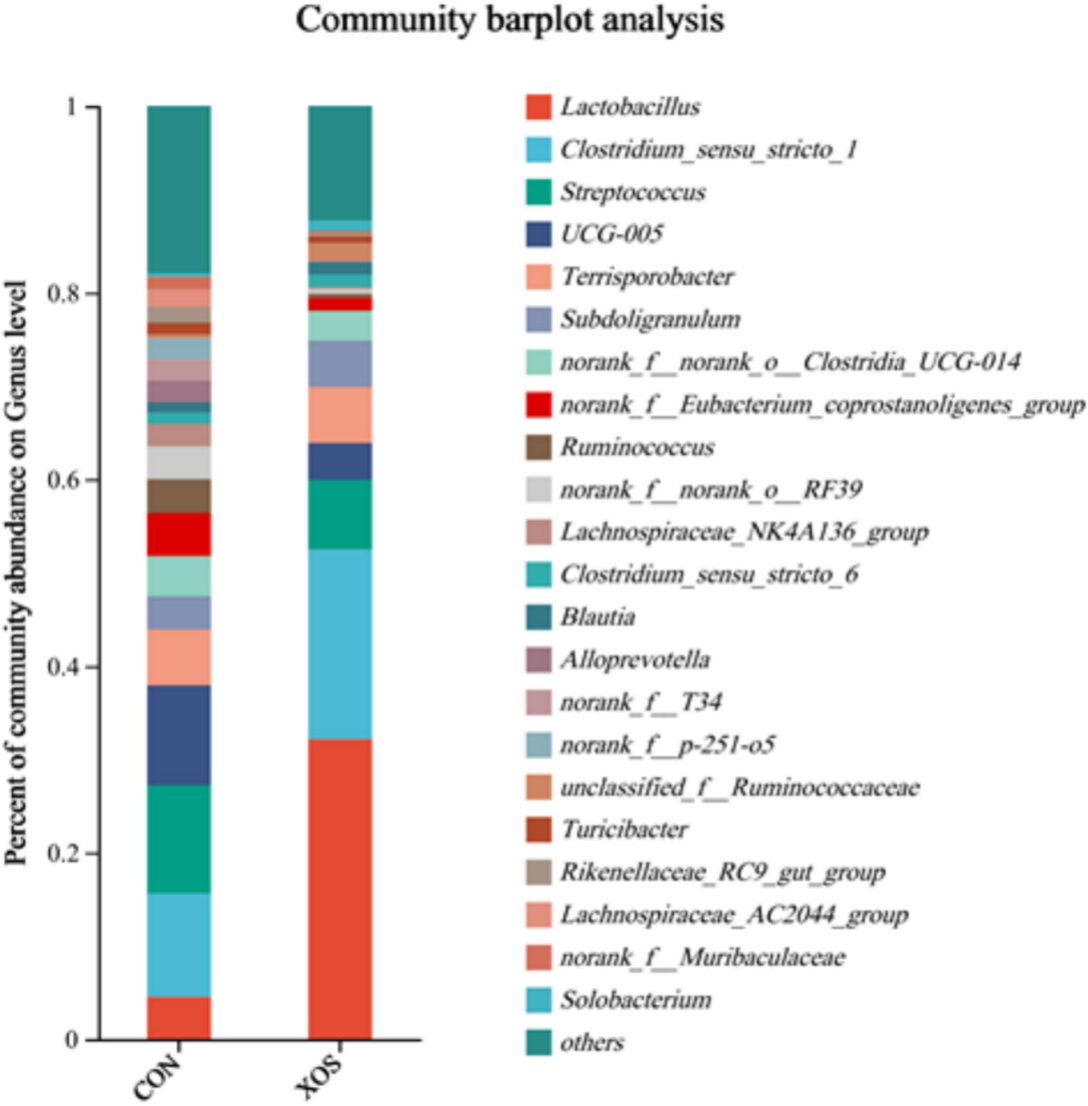
Effects of XOS on the composition of cecal microbial flora at genus level in weaned pigs.

**Figure 5:**
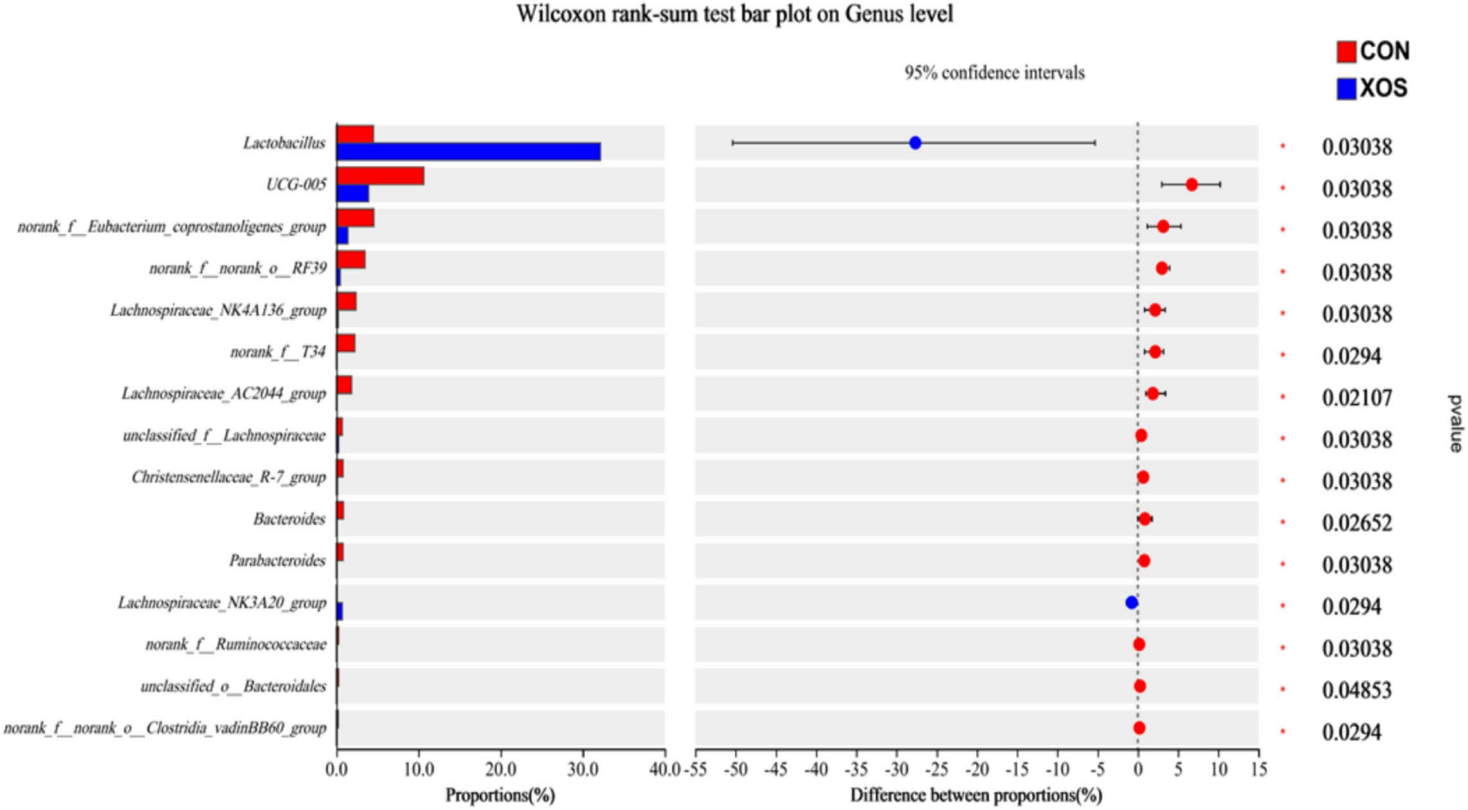
Relative abundance of different bacteria at the genus level.

As shown in Figures 4, 5 and Table 8, a total of 161 genera were identified, mainly consisting of *Lactobacillus*, *Clostridium_sensu_stricto_1*, *Streptococcus*, *UCG-005*, *Terrisporobacter*, *Subdoligranulum*, *Ruminococcus*, *Lachnospiraceae_NK4A136_group*, and *Alloprevotella*. Thirteen genera are represented. The relative abundance of *Lactobacillus* and *Ruminococcus* was significantly higher and the relative abundance of *Lachnospiraceae_NK4A136_group*, *Alloprevotella* and *Alloprevotella_AC2044_group* was significantly decreased compared with the XOS group and the control group (*p* < 0.05). Notably, the abundance of *Lactobacillus* increased by 274.94%.

**Table 8:** Effects of XOS on the composition of cecal microbial flora at genus level in weaned pigs.

Items	CON	XOS	SEM	*p-*value
*Lactobacillus*	8.54^b^	32.02^a^	0.88	0.01
*Clostridium_sensu_stricto_1*	13.16	18.95	2.93	0.92
*Streptococcus*	13.57	9.41	3.24	0.10
*UCG-005*	12.68	8.06	2.81	0.11
*Terrisporobacter*	5.99	4.39	0.85	0.46
*Eubacterium_coprostanoligenes*	4.57	4.57	0.12	0.24
*Subdoligranulum*	3.55	4.31	0.63	0.75
*Clostridia_UCG-014*	4.32	3.18	0.55	0.24
*Ruminococcus*	0.41^b^	5.37^a^	1.21	0.01
*Lachnospiraceae_NK4A136_group*	2.39^a^	0.51^b^	1.01	0.04
*Alloprevotella*	2.32^a^	0.13^b^	0.96	0.02
*Lachnospiraceae_AC2044_group*	1.86^a^	0.99^b^	0.82	0.04

Mean values within a same row between a and b were significantly different when *P* < 0.05, *n* = 6.

## 4 Discussion

Several studies have shown that XOS is a kind of functional prebiotic and has the potential effect to improve growth performance of pigs (1, 16). In this study, it was found that the addition of 500 mg/kg XOS significantly reduced the feed to gain ratio, but no difference was detected in ADG and ADFI. However, the effects of XOS on the growth performance of piglets are not completely consistent in several reports. Ding et al. (17) found that dietary addition of 250 mg/kg XOS increased the ADG and ADFI of weaned pigs, and reduced the feed to gain ratio. Ding et al. (17) also obtained an improvement in performance and the facilitation of intestinal development in piglets. However, Sun et al. (18) showed that adding 1 g/kg XOS to the diet had no effect on the ADG and ADFI for weaned piglets, which was similar to the results of this present study. We speculated that the reason for the inconsistency of these results may be related to the changes in the intestinal microbiota (19). Dietary XOS supplementation is thought to improve the nutrient utilization efficiency of animals. Lin et al. (20) found that dietary XOS supplementation improves the ileal nutrient digestibility, apparent metabolizable energy, and intestinal total mineral metabolism of broilers. Zhou et al. (21) found that dietary XOS supplementation improves the dietary nutrient digestibility and ileal morphology of laying hens. Few reports were concerned about the nutrient utilization efficiency of XOS in weaned pigs. In this experiment, the apparent digestibility of EE and Ca significantly increased after dietary 500 mg/kg XOS supplementation. The addition of XOS promoted the proliferation of intestinal lactobacilli bacteria, thereby establishing an acidic environment in the intestine, and facilitated the improvement of the absorption efficiency of EE and *Ca.*

Serum alkaline phosphatase (ALP) is mainly derived from the liver and bone. It can improve the absorption and transport of nutrients and some enzymes, and it can improve the feed conversion rate (22). In this study, the dietary supplementation of 500 mg/kg XOS increased the serum ALP activity of weaned pigs. Thus, XOS may improve the metabolic capacity of liver and promote the absorption and utilization efficiency of nutrients in pigs.

Several results suggested that XOS can improve intestinal morphology in animals (23). An improvement in gut morphology was observed when 100 or 500 mg/kg XOS was added to the diets of piglets or broilers, respectively (24, 25). The results of the present study found that the dietary addition of 500 mg/kg XOS increased villus height of the duodenum and ileum, which was consistent with the results of previous studies. The improvement of gut morphology by XOS in weaning piglets may also be related to its role as a kind of prebiotic in feed (1, 17). Some studies demonstrated that the intestinal flora of piglets can utilize XOS and produce a large amount of short-chain fatty acids such as butyric acid, propionic acid, and acetic acid, which can reduce intestinal pH, inhibit the proliferation of pathogenic bacteria to protect the intestinal mucosa and improve the intestinal morphology of weaned pigs (26, 27).

During the weaning stage, piglets are subjected to environmental stress, which may lead to the negative effects on antioxidant status. The enzymes activity of SOD and CAT play a crucial role in maintaining the normal antioxidant balance in weaning pigs. The MDA is a marker of oxidative stress, and its accumulation also directly reflects the degree of oxidative damage of animals. Li et al. (28) found that dietary XOS supplementation increases the total antioxidant capacity of broilers at 24 and 42 days of age. Yang et al. (25) also found that dietary supplementation of XOS in broilers improves total serum antioxidant capacity and muscle SOD activity. Pang et al. (13) reported a reduction of MDA in serum and increase of GSH-Px activity by adding 0.75, 1.5%, or 3% XOS to the diets of mice. The results in this present experiment were consistent with the above studies, and the addition of XOS increased the antioxidant enzyme activity in serum, liver, spleen and jejunum. It might indicate that the 500 mg/kg XOS can effectively improve the antioxidant capacity for pigs during weaning period.

The intestinal flora plays a crucial role in sustaining the normal physiological functions of the intestine, also impacting the absorption of nutrients and the immune barrier in the pig’s intestinal tract (29). The cecal flora is mainly composed of *Firmicutes* and *Bacteroidetes*, and *Lactobacillus* is the dominant bacteria in *Firmicutes* (30). The results of this study were basically consistent with the composition of cecal bacteria and dominant bacteria genera mentioned above. In this study, dietary supplementation of 500 mg/kg XOS changed the relative abundance of *Firmicutes* and *Bacteroides* in cecum microflora; the relative abundance of *Firmicutes* increased, whereas the relative abundance of *Bacteroides* and *Proteobacteria* decreased. Yin et al. (31) reported that dietary supplementation of 250 mg/kg XOS improves the relative abundance of *Bacteroidetes* and decreases the relative abundance of *Proteobacteria* in the gut of weaned pigs. *Lactobacillus* is the dominant genus of *Firmicutes*. It is also an important beneficial bacteria in the cecum, which has an impact on animal health, including producing beneficial metabolites, participating in immune regulation, and eliminating pathogenic bacteria and other functions. In this study, the addition of 500 mg/kg XOS increased the relative abundance of *Lactobacillus* in cecum, and this conclusion was consistent with the results of other studies. XOS has the potential effect to promote the proliferation of beneficial bacteria in cecum, and the increase in *Lactobacillus* abundance may help improve intestinal morphology and promote the growth of weaned pigs. Yuan et al. (32) also reported that *Lactobacillus* LR1 increases the ratio of villus height to crypt depth in the ileum of weaned piglets, which also supported the results of the present experiment. In addition, we observed a positive correlation between the increasing number of lactic acid bacteria and weight gain, which was confirmed in other studies (33, 34). Zeng et al. (35) reported that stress conditions could cause disorders in the microbial community, but the composition of microorganisms could be regulated by the nutritional method. In addition, the current study revealed a significant elevation in the relative abundance of *Ruminococcus* in the cecal microbiota of weaned pigs following the supplementation of 500 mg/kg XOS. *Ruminococcus*, a member of the phylum *Firmicutes*, possesses the ability to degrade dietary cellulose while also fermenting glucose and oligosaccharides (36). This increase in the relative abundance of *Ruminococcus* may be attributed to the provision of nutrients from the cellulose and hemicellulose components present in XOS, thereby fostering the growth and proliferation of *Ruminococcus* bacteria (36). The present study also observed reductions in the relative abundance of *Proteobacteria* and *Spirochetes* in the microbial composition of the cecum, both of which were considered harmful bacteria for weaned pigs (37). The increase in the relative abundance of *Proteobacteria* easily leads to inflammation, and its increase can be used as a signal of disease (38). And the *Spirochetes* and their members may also cause diseases such as leptospirosis, Lyme disease, and recurrent fever (39).

## 5 Conclusion

The results of this study showed that dietary supplementation of 500 mg/kg XOS could reduce the ratio of feed to gain of weaned pigs, improve the small intestine’s morphological structure, increase the apparent digestibility of EE and Ca, and enhance serum ALP activity and the antioxidant capacity of weaned pigs. The addition of XOS increased the relative abundance of beneficial bacteria such as *Lactobacillus* and optimized the flora structure.

## Data Availability

The original contributions presented in the study are included in the article/Supplementary material. Further inquiries can be directed to the corresponding author(s). Sequences were deposited in the NCBI database under accession number PRJNA1321560.
