# Supplementary material for: Effects of xylooligosaccharide supplementation on performance, intestinal morphology, antioxidant capacity and cecal microflora in weaning pigs
Source: Front Vet Sci. 2025 Sep 17;12:1655379. doi: 10.3389/fvets.2025.1655379 (PMC12483880; doi:10.3389/fvets.2025.1655379)
Supplement: Supplementary file 1 [file Table_1.DOCX]

# Alpha diversity analysis

As shown in Table 1, the addition of 500 mg/kg XOS to the diet had no significant effect on the Shanon index, Simpson index, Chao index and Sobs index of cecum microorganisms in weaned pigs.

**Table 1. The Alpha diversity index in the cecum microorganism.**

| **Items** | **CON** | **XOS** | **SEM** | ***P-*value** |
| --- | --- | --- | --- | --- |
| Shanon index | 3.28 | 2.92 | 0.12 | 0.45 |
| Simpson index | 0.08 | 0.14 | 0.08 | 0.58 |
| Chao index | 285.99 | 307.95 | 21.70 | 0.23 |
| Sobs index | 253.50 | 258.67 | 15.21 | 0.59 |
